# The influence of short sprint performance, acceleration, and deceleration mechanical properties on change of direction ability in soccer players—A cross-sectional study

**DOI:** 10.3389/fphys.2022.1027811

**Published:** 2022-11-02

**Authors:** Qingshan Zhang, Alexandre Dellal, Karim Chamari, Pierre-Hugues Igonin, Cyril Martin, Christophe Hautier

**Affiliations:** ^1^ School of Athletic Performance, Shanghai University of Sport, Shanghai, China; ^2^ Laboratoire Interuniversitaire de Biologie de la Motricité, Université de Lyon, UCBL1, UFRSTAPS, Villeurbanne Cedex, France; ^3^ FIFA Medical Centre of Excellence, Centre orthopédique Santy, Sport Science and Research Department, Lyon, France; ^4^ Mycoach Pro & Mycoach Performance, Performance department, Nice, France; ^5^ Aspetar, Orthopaedic and Sports Medicine Hospital, FIFA Medical Centre of Excellence, Doha, Qatar; ^6^ ISSEP Ksar-Said, La Manouba University, Tunis, Tunisia; ^7^ Association Sportive de Saint-Etienne (ASSE), Saint-Etienne, France

**Keywords:** sprint, propulsive force, braking force, COD, football

## Abstract

The study investigated the relationship between short sprint performance and mechanical parameters obtained during the acceleration and deceleration tasks with the change of direction (COD) performance in female and male soccer players. The acceleration and deceleration ability were compared in the “High/Fast” *versus* “Low/Slow” COD performance group based on a median split analysis in each sex group. One hundred three French soccer players were assessed for the sprinting Force-Velocity (F-V) profile (i.e., theoretical maximal force [F0], velocity [V0], power [Pmax]), 10 m performance, linear deceleration test (maximal braking force [HBF_max_], braking power [BP_max_], deceleration [Dec_max_]), and COD performance using 505-test. The 10 m performance was strongly associated with 505-test performance (ES = [0.64 to 0.71]), whereas the sprinting F-V profiles parameters were weakly to moderately correlated with 505- performance (ES = [-0.47 to -0.38]). The BP_max_ was also moderately associated with 505-test performance (ES: range = [-0.55 to -0.46]). In addition, the High/Fast female COD group presented higher F0, Pmax, HBF_max_, and BP_max_ than the Low/Slow group, whereas the male groups presented very few mechanical differences. Multiple regression analysis shows that the COD performance of male players was determined by 10 m performance and maximum deceleration power. In contrast, no statistically significant model could be found to determine the change of direction performance in female players. In conclusion, the current finding indicated that the only variable strongly associated with COD performance was the linear 10 m sprint time. In the same way, the mechanical parameters obtained from acceleration and deceleration seemed to play a non-neglectable role in this population.

## Introduction

Change of direction (COD) while sprinting at high speed is essential in most team sports, such as soccer or rugby. To date, the optimal COD performance has been demonstrated to be influenced by numerous factors, including the sprint performance (Sayers, 2015), lower limb strength (Jones et al., 2017; Harper et al., 2021), reactive strength (Castillo-Rodriguez et al., 2012), body stability (Sasaki et al., 2011) and braking technique (Brughelli et al., 2008). Evaluation of COD performance can be done with different tests. Still, one of the most commonly used tests is the 505-test, which consists of four phases: an initial acceleration, a deceleration, a 180-degree COD, and a re-acceleration. Still, athletes can have widely varying abilities in the different components of these explosive capacities.

Acceleration during running requires the athlete to move their body forward rapidly while producing the highest horizontal propulsion force. Previous studies have shown that acceleration ability correlates with performance on the 505-test (Little and Williams, 2005; Buchheit et al., 2014; Loturco et al., 2019; Baena-Raya et al., 2021a). For example, Loturco et al. found that athletes with better COD performance exhibited shorter 5-m sprint times (Loturco et al., 2019). Recently, Baena et al. (2021) demonstrated that the variable that displayed a strong association with COD performance was short sprint time (10m, r > 0.7) (Baena-Raya et al., 2021b). In addition, considering that propulsive force may play a critical role in determining acceleration capacity, Baena-Raya et al.(Baena-Raya et al., 2021a) investigated the influence of sprint mechanical properties assessed by the Force-Velocity (F-V) profile on 505-test performance. They reported that maximum power (P_max_) (r = -0.82) and velocity (V0) (r = -0.76) of sprinting were strongly correlated with 505-test performance in female futsal players. Additionally, while there is a large amount of literature on the kinematics and kinetics of the acceleration phase in sprinting and the links to the F-V relationship have been extensively studied (Samozino et al., 2022), the determinants of the deceleration have been less studied. The deceleration task is employed to rapidly stop or decrease the body’s center of mass to prepare for the change of direction movement prior to the re-acceleration (Dos'Santos et al., 2017; Jones et al., 2017). The athlete must produce high braking forces to decelerate quickly. Jones et al.(Jones et al., 2017) measured large ground reaction forces during the penultimate and final foot contact before the change of direction (i.e., the 505-test) in female soccer players. Their results indicate that players producing the highest braking force have the best COD performance because it allows them to make a faster transition between the end of braking (e.g., penultimate and earlier foot contacts) and the re-acceleration (Spiteri et al., 2015). In addition, some previous research has indicated that the deceleration deficit is essential in determining the 505-test performance (Clarke et al., 2020). Although the above findings suggest that deceleration capacity is a critical factor in COD performance, our understanding of the association between deceleration capacity and COD performance is not extensive to date. Studying the braking force and power production during the deceleration phase should make it possible to characterize better the mechanical properties essential to high COD performance in this field. Furthermore, it has been shown that male athletes performed better COD performances and higher sprint F-V mechanical properties than female athletes (Zhang et al., 2021b; Freitas et al., 2021); nonetheless, few studies have investigated the influence of the mechanical properties obtained from the deceleration in different sexes. As a result, it is also essential to determine whether sex differences induce the special relationship between acceleration and deceleration abilities and COD performance based on the differences in physical skills and match activity profiles reported between the male and female players.

Therefore, the purpose of the present study was to 1) determine the relationships between acceleration and deceleration capabilities and COD performance, 2) compare acceleration and deceleration capabilities in athletes with High *versus* Low COD performance, and 3) attempt to build a model of COD performance in female and male soccer players. We hypothesized that COD performance 1) would be influenced by acceleration and deceleration force and power, 2) the High COD performance represents better acceleration and deceleration capabilities, and 3) the predictive COD performance models would differ between male and female soccer players.

## Materials and methods

### Experimental approach

The present study used a cross-sectional design to investigate the relationship between acceleration, deceleration, and COD performance in team sports players. The protocol consisted of experimental testing sessions, including acceleration, linear deceleration, and the 505 test in random order. All participants performed a standardized 20-min dynamic warm-up protocol on the field-specific to soccer. After ∼6 min of recovery, participants performed in random order two accelerations, two decelerations, and two 505-tests from a crouched position (staggered stance) with a recovery period of 4 min and 10 min between each trial and each test, respectively.

### Participants

One hundred and three French amateur/semi-professional athletes (53 males and 50 females, height: 1.73 ± 1.1 vs. 1.66 ± 0.01 cm, mass: 70.48 ± 9.0 vs. 58.76 ± 6.88 kg, age: 19.04 ± 2.05 vs. 20.09 ± 3.24 years, training volume: 4.1 ± 1.0 vs. 7.3 ± 3.39 h week-1, training experience: 10.96 ± 1.77 vs 11.83 ± 3.23 years) volunteered to participate in this study. Subjects had been training at least four times per week for more than 8 years. The *a priori* sample size (n = 100) was calculated using G-Power 3.1(Brunsbuttel, Germany) in the basic ANOVA test, which assumes a large effect size f = 0.4, error *α* = 0.05, and 1-β = 0.95. All participants had no lower extremity injuries in the past 12 months. After being informed of the procedure, all participants gave written informed consent to participate in the protocol. Prior to the experiments, the participants followed their usual training program but did not perform any intense workouts or unusual matches 48 h before the protocol. The study was approved by the “XXX” ethics committee of XXX. All participants performed one session of habituation testing (sprint, deceleration, and COD running) and one session of maximal testing during the regular training session to decrease the effects of the circadian cycle. Participants performed sprinting, deceleration, and COD tests during the testing session.

## Experimental sessions

### Maximal acceleration test

A 30 m maximum sprint test assessed maximum acceleration capabilities. A Stalker Acceleration Testing System (ATS) II radar device (Stalker ATS II, Applied Concepts, Dallas, TX, United States, 46.9 Hz) was attached to a sturdy tripod placed 5 m behind the starting line at the height of 0.9 m above the ground (corresponding approximately to the height of the subject’s center of mass) to record the speed-time curve during the sprint. Intermediate sprint times were recorded over 5 m and 10 m) using timing cells (Witty, Microgate^®^, Bolzano, Italy) set at the same height as the radar device. Athletes were encouraged to sprint “through” each distance marker (i.e., 5 m, 10 m, 15 m, 20 m, 25 m, 30 m) to ensure a complete maximum collection without deceleration. The mechanical properties of sprinting were assessed through the mechanical parameters of the Force-Velocity profile (i.e., F0, V0, P_max_) according to the validated method of Samozino (Samozino et al., 2016).

### Maximal deceleration test

Maximum deceleration performance was assessed by the 20 m linear deceleration test (Zhang et al., 2021a). Participants first began running in a maximal 20 m sprint. They were then instructed to stop as quickly as possible after the 20 m sprint (e.g., braking line) and return to the 20 m line by backpedaling. This created a clear change in velocity on the instantaneous velocity-time graph captured by the radar device and enabled the end of the deceleration phase to be easily identified (Harper et al., 2020). The start of the deceleration phase was defined as the moment of maximal velocity achieved during the 20 m sprint running. Furthermore, the end of the deceleration phase was defined as the lowest velocity following maximal velocity. Any 20 m time 5% slower than the best 20 m split time obtained during the sprint test was not considered for analysis. The radar device, as mentioned above, was used to record the speed-time curve during the deceleration phase. Kinetic and kinematic variables, including horizontal braking force (HBF), braking power (BP), and deceleration (Dec) between the start and end of the deceleration phase, were calculated according to the previously validated method based on Newton’s second law of motion (Harper et al., 2020). The maximal braking force (HBF_max_), power (BP_max_), and deceleration (Dec_max_) were obtained as the highest value of all instantaneous HBF, BP, and Dec values during the entire deceleration phase for data analysis.

### 505-test

The COD performance was assessed using the 505 change of direction test on the left side (COD_L) and right side (COD_R). Participants started the test 0.5 m behind the starting line, and the turning line was set at 15 m from the start line. A pair of timing gates (MicroGate Witty Timer System, Bolzano, Italy) was placed at 10 m in the front of the starting line, set at approximately hip height. The participants were instructed to accelerate as fast as possible to reach the turning line, then place the left or right foot on the turning line to turn around 180° and run back as quickly as possible for 5 m (Barber et al., 2016). The COD performance was evaluated as the time to go from and return to the 10 m line.

### Statistical analysis

Before performing the statistical analysis, the Shapiro–Wilk test was used to assess the normality of the data. The correlation (r) between the target variable of acceleration, deceleration, and COD performance was calculated using Pearson’s or Spearman’s correlation analysis with Holm correction. The magnitude of the correlation coefficient (r) was interpreted using criteria: very weak (0.11–0.19), weak (0.20–0.39), moderate (0.40–0.59), strong (0.60–0.79), and very strong (0.80–1.00). Within each sex group, players were also classified as High/Fast and Low/Slow using a median split based on the better COD performance to examine acceleration and deceleration qualities further. A linear mixed model was then used to evaluate the fixed effect of gender (Male vs Female) and the COD qualities (High vs Low) with the random intercepts as a between-subject factor on the mechanical properties of acceleration (F0, V0, P_max_, 10-m) and deceleration (HBF_max_, BP_max_, Dec_max_), respectively. The effect size was calculated using partial eta-squared (η2) to evaluate the magnitude of differences between the groups, giving the scale as small (0.01), medium (0.09), and large (0.25). Furthermore, a linear mixed-effects regression analysis was used to test the relationship between acceleration performance (fixed effect) and the corresponding deceleration mechanical parameters (fixed effects) as independent variables with the random intercepts as between-participants factors and COD performance as dependent variables. The effect size was calculated as $f^{2}=\frac{r^{2}}{1-r^{2}}$ , and interpreted using criteria: trivial (<0.02), low (0.02–0.15), medium (0.15–0.35) or high (>0.35). Within-test reliability was quantified using the intraclass correlation coefficient (ICC), coefficient of variation (CV), and standard error of the measurement (SEM). The ICC values were interpreted using criteria: excellent (>0.9), good (0.75–0.9), moderate (0.5–0.75), and poor (<0.5). The value of *p* was set at a 0.05 significance level. All statistical procedures were performed with R software (R 3.5.0, R Core Team, Vienna, Austria). Descriptive statistics are presented as mean ± SD with 95% CI.

## Results

**FIGURE 1:**
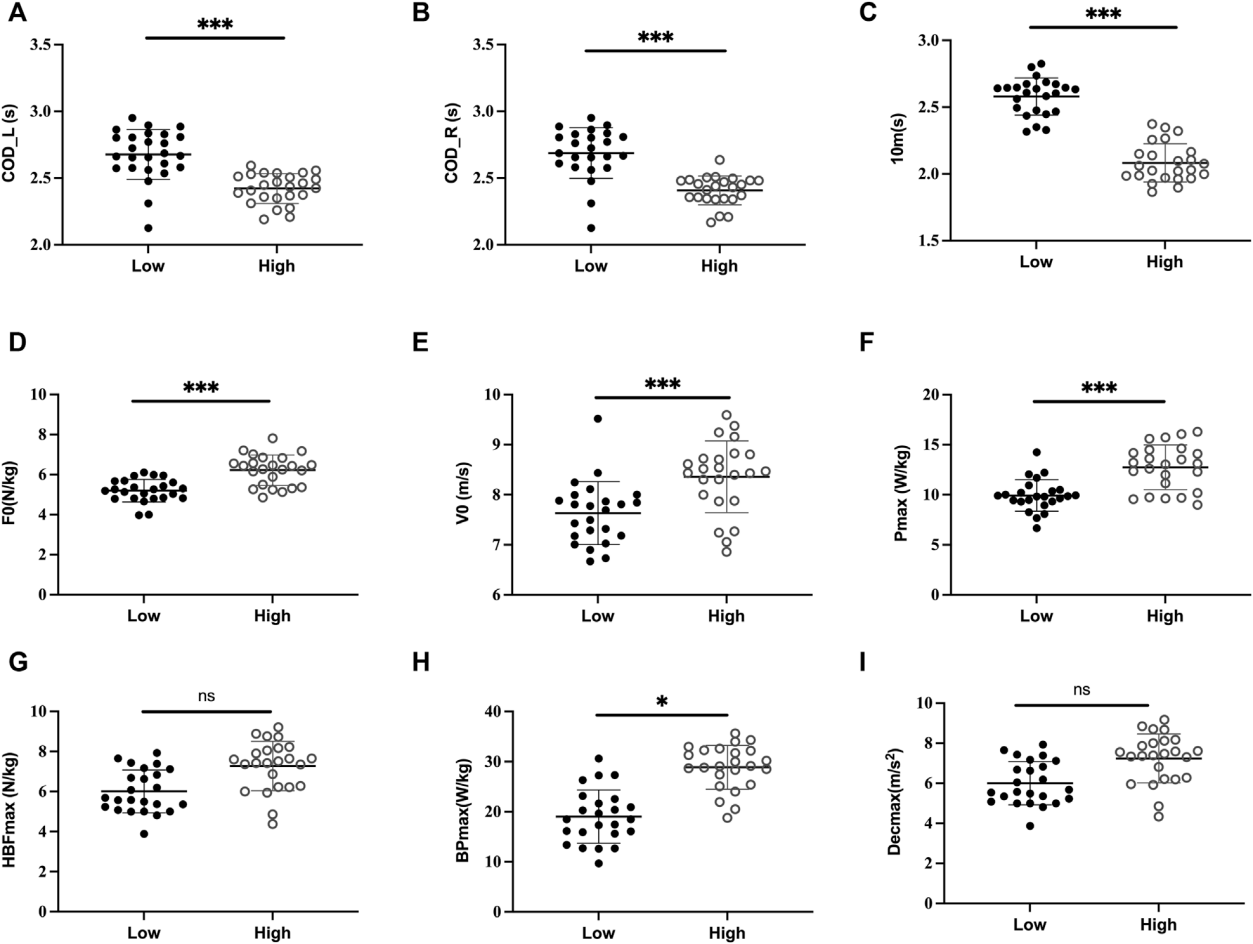
Acceleration and deceleration mechanical parameters and COD performance on the left side (COD_L) and right side (COD_R) are displayed by COD performance (High vs. Low) in female soccer players; F0: maximal theoretical force; V0: maximal theoretical velocity; P_max_: maximal power; 10 m: 10 m split time; HBF_max_: maximum braking force; HBP_max_: maximum braking power; Dec_max_: maximum deceleration; *: *p* < 0.05; **: *p* < 0.01; ***: *p* < 0.001; ns: non-significant.

**FIGURE 2:**
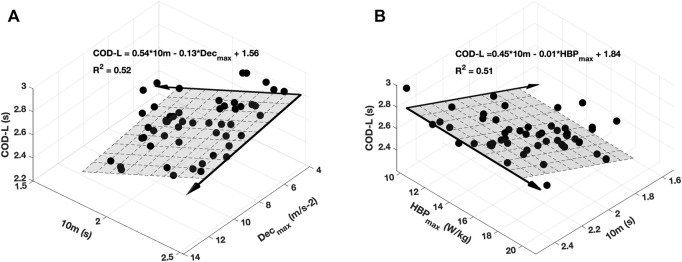
Acceleration and deceleration mechanical parameters and COD performance on the left side (COD_L) and right side (COD_R) are displayed by COD performance (High vs. Low) in male soccer players; F0: maximal theoretical force; V0: maximal theoretical velocity; P_max_: maximal power; 10 m: 10 m split time; HBF_max_: maximum braking force; HBP_max_: maximum braking power; Dec_max_: maximum deceleration; *: *p* < 0.05; **: *p* < 0.01; ***: *p* < 0.001; ns: non-significant.

The association between 10 m sprint performance, acceleration and deceleration ability, and COD performance were presented in Tables 1, 2. The 10 m sprint performance, acceleration, and deceleration ability, and COD performance were presented in Table 4 and Figures 1, 2. The COD performance, 10-m sprint time, and mechanical parameters of acceleration and deceleration indicated good to excellent reliability (ICC = 0.75 ∼ 0.95) with low variability (CV = 0.3 ∼ 2.6) (Table 1).

**TABLE 1:** Intra Coefficient Correlation (ICC) for the COD performance, 10-m sprint time, and mechanical parameters of acceleration and deceleration. ICC = intraclass correlation coefficient; CV = coefficient of variation; SEM = standard error of the measurement.

		Female			Male		
		ICC	CV%	SEM	ICC	CV%	SEM
Acceleration	F0	0.91 [ 0.74; 0.97 ]	1.1	0.13	0.86 [ 0.6; 0.95 ]	0.9	0.12
	V0	0.84 [ 0.55; 0.95 ]	0.5	0.08	0.79 [ 0.44; 0.93 ]	0.6	0.1
	Pmax	0.92 [ 0.76; 0.97 ]	1.4	0.35	0.88 [ 0.67; 0.96 ]	1.1	0.31
	10-m	0.95 [ 0.83; 0.98 ]	0.5	0.02	0.81 [ 0.48; 0.94 ]	0.4	0.02
Deceleration	HBFmax	0.89 [ 0.66; 0.97 ]	1.5	0.24	0.9 [ 0.71; 0.97 ]	2.2	0.35
	BPmax	0.79 [ 0.41; 0.93 ]	1.3	0.77	0.8 [ 0.46; 0.93 ]	1.3	0.67
	Decmax	0.89 [ 0.66; 0.97 ]	1.5	0.25	0.95 [ 0.84; 0.98 ]	2.6	0.39
COD	505-R	0.82 [ 0.49; 0.94 ]	0.5	0.03	0.75 [ 0.34; 0.92 ]	0.3	0.01
	505-L	0.84 [ 0.57; 0.95 ]	0.4	0.02	0.76 [ 0.36; 0.93 ]	0.4	0.02

**TABLE 2:** Associations between acceleration and deceleration mechanical parameters and COD performance on left side (COD_L) and right side (COD_R) for female players. F_0_: maximal theoretical force; V_0_: maximal theoretical velocity; P_max_: maximal power; 10 m: 10 m split time. HBF_max_: maximum braking force; HBP_max_: maximum braking power; Dec_max_ maximum deceleration.

			Correlation coefficient, r (95 CI%) (95% CI)	Qualitative inference	*p*-value
		Variable			
**COD_L**	**Acceleration**	F_0_ (N·kg^−1^)	-0.38 [ -0.6; -0.12 ]	Weak	0.01
		V_0_ (m·s^−1^)	-0.22 [ -0.47; 0.06 ]	Weak	0.12
		P_max_ (W·kg^−1^)	-0.33 [ -0.55; -0.06 ]	Weak	0.02
		10 m	0.62 [ 0.42; 0.77 ]	Strong	<0.001
	**Deceleration**	HBF_max_ (N.kg^−1^)	-0.42 [ -0.62; -0.16 ]	Moderate	<0.001
		BP_max_ (W.kg^−1^)	-0.48 [ -0.67; -0.23 ]	Moderate	<0.001
		Dec_max_ (m.s^−2^)	-0.42 [ -0.62; -0.16 ]	Moderate	<0.001
**COD_R**	**Acceleration**	F_0_ (N·kg^−1^)	-0.47 [ -0.66; -0.22 ]	Moderate	0.01
		V_0_ (m·s^−1^)	-0.34 [ -0.56; -0.07 ]	Weak	0.01
		P_max_ (W·kg^−1^)	-0.44 [ -0.64; -0.19 ]	Moderate	<0.001
		10 m	0.67 [ 0.49; 0.8 ]	Strong	<0.001
	**Deceleration**	HBF_max_ (N.kg^−1^)	-0.36 [ -0.58; -0.1 ]	Weak	0.01
		BP_max_ (W.kg^−1^)	-0.48 [ -0.67; -0.24 ]	Moderate	<0.001
		Dec_max_ (m.s^−2^)	-0.36 [ -0.58; -0.09 ]	Weak	0.01

### Correlation between acceleration, deceleration, and COD performance

F0 and P_max_ showed a weak to moderate negative (-0.47 < *r* < -0.38; *p* < 0.05) association with COD performance of right and left side in female soccer players (Table 2). The V0 had a weak association with the COD_R performance (r = 0.34; *p* = 0.01), whereas no significant correlation was found between V0 and COD_L performance in female soccer players (Table 2, Figure 1). Additionally, V0 and P_max_ had a moderate negative correlation with the COD-test performance (-0.47 < *r* < -0.40; *p* < 0.001), whereas no association was found between F0 and 505-test performance, whatever the direction in male soccer players (Table 3, Figure 2). Notably, 10-m split time strongly correlated with 505 performances (all *p* < 0.001) irrespective of the direction and gender (Table 2, 3). All the deceleration mechanical parameters had moderate association with the COD_L performance (-0.5 < *r* < -0.41; *p* < 0.001) in female soccer players (Table 2). Only BP_max_ had a moderate correlation with the COD_R (*p* < 0.001), whereas both HBF_max_ and Dec_max_ had a weak association (r ≈ -0.35; *p* = 0.01) with the COD_R performance in female soccer players (Table 2). In male soccer players, only BP_max_ had moderate association with the COD_L (*p* < 0.001) and COD_R performances (*p* < 0.001) in both directions (Table 3). In contrast, no significant association was found between the HBF_max_, Dec_max_ and COD performances (all *p* > 0.05, Table 3).

**TABLE 3:** Associations between acceleration and deceleration mechanical parameters and COD performance on the left side (COD_L) and right side (COD_R) for male players. F_0_: maximal theoretical force; V_0_: maximal theoretical velocity; P_max_: maximal power; 10 m: 10 m split time; HBF_max_: maximum braking force; HBP_max_: maximum braking power; Dec_max_: maximum deceleration.

		Variable	Correlation coefficient, r (95 CI%) (95% CI)	Qualitative inference	*p*-value
**COD_L**	**Acceleration**	F_0_ (N·kg^−1^)	-0.23 [ -0.47; 0.05 ]	Weak	0.1
		V_0_ (m·s^−1^)	-0.48 [ -0.66; -0.24 ]	Moderate	<0.001
		P_max_ (W·kg^−1^)	-0.45 [ -0.64; -0.2 ]	Moderate	<0.001
		10-m	0.69 [ 0.52; 0.81 ]	Strong	<0.001
	**Deceleration**	HBF_max_ (N.kg^−1^)	-0.21 [ -0.46; 0.06 ]	Weak	0.13
		BP_max_ (W.kg^−1^)	-0.54 [ -0.71; -0.31 ]	Moderate	<0.001
		Dec_max_ (m.s^−2^)	-0.21 [ -0.45; 0.07 ]	Weak	0.14
**COD_R**	**Acceleration**	F_0_ (N·kg^−1^)	-0.22 [ -0.46; 0.06 ]	Weak	0.12
		V_0_ (m·s^−1^)	-0.43 [ -0.63; -0.17 ]	Moderate	<0.001
		P_max_ (W·kg^−1^)	-0.40 [ -0.61; -0.15 ]	Moderate	<0.001
		10-m	0.70 [ 0.53; 0.82 ]	Strong	<0.001
	**Deceleration**	HBF_max_ (N.kg^−1^)	-0.12 [ -0.38; 0.16 ]	Very weak	0.42
		BP_max_ (W.kg^−1^)	-0.45 [ -0.64; -0.2 ]	Moderate	<0.001
		Dec_max_ (m.s^−2^)	-0.11 [ -0.37; 0.17 ]	Weak	0.42

### Differences in acceleration and deceleration performance between High/Fast and Low/Slow COD groups

The High/Fast COD group showed a higher 10-m split time and mechanical parameter of acceleration and deceleration with larger effect size (0.24 < η2 < 0.62; *p* < 0.001), except for Dec_max_ (η2 = 0.05; *p* = 0.02) compared to Low COD group in female soccer players (Table 4; Figure 1, 2). In contrast, High COD male group only showed higher 10-m performance and P_max_ (η2 = 0.08; *p* = 0.02) and BP_max_ (η2 = 0.12; *p* < 0.001) compared to the Low COD group (Table 4; Figure 1, 2).

**TABLE 4:** Kinematic and kinetic variables during the acceleration, deceleration, and COD performance on the left side (COD_L) and right side (COD_R) were displayed by gender (Male vs. Female) and COD performance (High vs. Low), and the comparison by the COD performance. F_0_: maximal theoretical force; V_0_ : maximal theoretical velocity; P_max_ : maximal power; 10 m: 10 m split time; HBF_max_: maximum braking force; HBP_max_: maximum braking power; Dec_max_: maximum deceleration. All data are presented as mean ± standard deviation with a 95% confidence interval. * Significant difference from COD level.

Gender	Variable	High/Fast		Low/Slow		Difference	p-value	Effect size(η2)	Qualitatie inference
		Mean ± SD	[95% CI]	Mean ± SD	[95% CI]				
Female (n = 50), High (n = 25), Low (n = 25)	COD_L (s)	2.42 ± 0.11	[0.09; 0.15]	2.67 ± 0.18	[0.12; 0.29]	0.26[0.17;0.34] *	<0.001	0.28	Large
	COD_R (s)	2.41 ± 0.11	[0.08; 0.16]	2.68 ± 0.19	[0.13; 0.3]	0.27[0.18; 0.36] *	<0.001	0.32	Large
	F0 (N·kg−1)	6.22 ± 0.76	[0.61; 1.04]	5.16 ± 0.55	[0.43; 0.77]	-1.06[-1.44;-0.68] *	<0.001	0.35	Large
	V0 (m·s−1)	8.36 ± 0.72	[0.55; 1.02]	7.7 ± 0.65	[0.47; 0.97]	-0.66[-1.05;-0.27] *	<0.001	0.32	Large
	Pmax (W·kg−1)	12.74 ± 2.24	[1.86; 2.94]	9.92 ± 1.51	[1.05; 2.37]	-2.82[-3.92;-1.72] *	<0.001	0.24	Large
	10 m(s)	2.08 ± 0.14	[0.11; 0.2]	2.55 ± 0.18	[0.13; 0.26]	0.46[0.37; 0.56] *	<0.001	0.62	Large
	Decmax (m.s−2)	7.24 ± 1.22	[0.91; 1.78]	6.2 ± 1.26	[1; 1.71]	-1.04[-1.74;-0.34]	0.13	0.05	Small
	HBFmax (N·kg−1)	7.28 ± 1.23	[0.92; 1.79]	6.21 ± 1.26	[0.99; 1.72]	-1.07[-1.78;-0.36]	0.11	0.06	Small
	BPmax (W.kg−1)	28.87 ± 4.39	[3.31; 6.35]	19.98 ± 6.16	[4.83; 8.49]	-8.89[-11.92;-5.86] *	0.02	0.27	Large
Male (n = 53), High (n = 26), Low (n = 27)	COD_L (s)	2.41 ± 0.11	[0.08; 0.15]	2.66 ± 0.16	[0.13; 0.22]	0.25[0.18; 0.33] **	<0.001	0.29	Large
	COD_R (s)	2.36 ± 0.11	[0.09; 0.16]	2.6 ± 0.14	[0.1; 0.21]	0.24[0.17; 0.31] *	<0.001	0.28	Large
	F0 (N·kg−1)	6.98 ± 0.7	[0.53; 1]	6.52 ± 0.69	[0.57; 0.9]	-0.46[-0.85;-0.08]	0.07	0.003	Small
	V0 (m·s−1)	9.44 ± 0.91	[0.63; 1.4]	8.92 ± 0.61	[0.43; 0.94]	-0.52[-0.95;-0.09]	0.056	0.08	Moderate
	Pmax (W·kg−1)	15.91 ± 1.4	[1.02; 2.07]	14.45 ± 1.82	[1.38; 2.58]	-1.46[-2.35;-0.57] *	0.02	0.08	Moderate
	10 m(s)	1.83 ± 0.08	[0.06; 0.11]	2.26 ± 0.1	[0.08; 0.15]	0.44[0.39; 0.48] *	0.01	0.6	Large
	Decmax (m.s−2)	7.71 ± 1.8	[1.37; 2.57]	7.27 ± 2.19	[1.69; 3.07]	-0.43[-1.54; 0.67]	0.94	0.01	Small
	HBFmax (N·kg−1)	7.74 ± 1.79	[1.36; 2.56]	7.34 ± 2.17	[1.7; 2.98]	-0.41[-1.5; 0.69]	0.11	0.05	Small
	BPmax (W.kg−1)	33.16 ± 3.96	[3.08; 5.5]	27.97 ± 5.65	[4.31; 7.97]	-5.19[-7.88;-2.51] *	<0.001	0.12	Moderate

### Linear mixed-effects regression analysis

**FIGURE 3:**
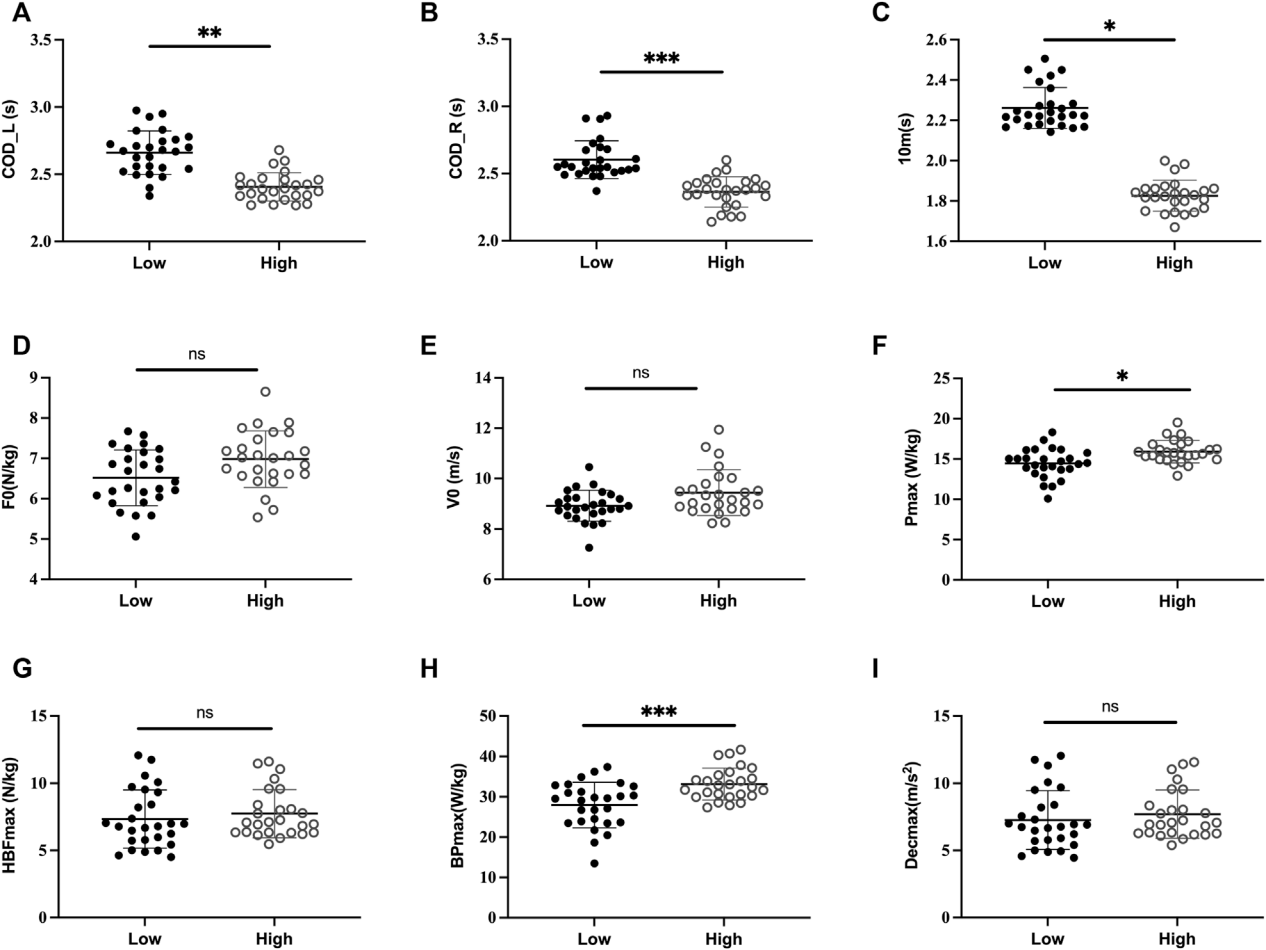
Two best multiple linear regressions for left side COD performance (COD_L) in male soccer players; 10 m: 10 m split time; HBP_max_: maximum braking power; Dec_max_: maximum deceleration.

Furthermore, the linear mixed-effects regression analysis showed a significant relationship between COD_L performance and the combination of 10-m and Dec_max_ (*r* ^2^ = 0.52; *f* ^2^ = 1.08; *p* < 0.001; Figure 3A), 10-m and HBF_max_ in male soccer players (*r* ^2^ = 0.53; *f* ^2^ = 1.13; *p* < 0.001; Figure 3B), whereas no significant model was found in female soccer players.

## Discussion

This study aimed to explore relationships between COD performance, 10 m sprint time, and the mechanical parameters of acceleration and deceleration in male and female soccer players. The main finding indicated that the 10-m sprint performance strongly correlates with COD performance in male and female players whereas the mechanical parameters of acceleration and deceleration presented weak to moderate correlations with COD performance.

### Relationship between COD performance, 10 m sprint time, and F-V mechanical characteristics

The present finding agrees with the previous studies reporting that the 10 m sprint performance is the primary variable strongly related to COD performance (Gabbett et al., 2008; Loturco et al., 2019). This could be explained by the short running distance performed during this test, so the propulsive phase (i.e., acceleration, re-acceleration phase) takes approximately 65% of the COD tasks (Nimphius et al., 2013). Most studies indicated that the superior linear accelerating capacity expressed in the 10 m sprinting contributed to the faster COD performance. As a result, the higher short sprint performance allows players to reach the deceleration line quickly, contributing to better COD performance. On the other side, it has been shown that the mechanical properties of sprinting reflect the athlete’s ability to effectively apply a high level of force to the ground to move the body in the forward direction. This may partly explain the weak to moderate correlations between F0 and P_max_ (Baena-Raya et al., 2021b) with COD performance. This study confirms the strong influence of 10 m sprint time on COD performance and suggested, it can be helpful to pay attention to the mechanical parameters obtained from sprinting to understand the COD performance better and improve this ability. This is particularly true considering the demonstrated influence of the kinetic parameters on the sprint performance (Hunter et al., 2005; Buchheit et al., 2014; Colyer et al., 2018; Nagahara et al., 2018).

Interestingly, a relationship was found between 10 m sprint time, maximal deceleration and braking power, and COD performance in male players. Numerous studies have proposed that the deceleration phase before changing the direction might be critical for COD performance (Dos'Santos et al., 2017; Dos'Santos et al., 2019; Thomas et al., 2020b). For instance, some recent studies reported that faster athletes in the 505-test displayed greater horizontal braking force (GRF) over the penultimate and last foot contact (Dos'Santos et al., 2019; Thomas et al., 2020b) in line with the present finding. The current result supports these prior investigations by demonstrating the association of linear deceleration mechanical properties with COD performance in male and female soccer players. However, a weak to moderate significant relationship was observed, indicating that other factors like technical skills, training content, and/or morphological particularities could impact COD performance more than force and power production in acceleration and deceleration phases. Additionally, it is also important to note that the requirement of physical and mechanical demands during a competitive match depends on the playing position, which might induce the different physical performance outputs for the specific playing position, including acceleration, deceleration, and COD profile. For instance, the midfielder decelerates more frequently than accelerates and has a 30% higher performance in sprinting than strikers and fullbacks (Di Salvo et al., 2007). Moreover, the midfielders showed a higher COD performance than the goalkeeper, whereas there was no significant difference in sprinting performance (e.g., 10 m) between them (Bujnovky et al., 2019). Thus, the playing position might contribute to the different output of deceleration ability following a similar COD performance and further studies are needed to explore this issue.

### Mechanical acceleration and deceleration properties of High/Fast and Low/Slow COD performance

The mixed-effects linear regression (Gender & COD performance) indicated that the players showed distinct mechanical properties of acceleration and deceleration. As reported in previous studies, male soccer players always display a shorter 10 m sprint time and higher sprinting mechanical parameters compared to female soccer players (Jimenez-Reyes et al., 2019; Zhang et al., 2021b). It seems that male players accelerated faster and applied higher GRF during sprint running compared to females (F0: ∼7.5 vs ∼6.7); this subsequently produced a faster sprint performance (Spiteri et al., 2013; Spiteri et al., 2014) (10-m performance: ∼1.9 s vs ∼2.3 s). On the other hand, male players do not have the same advantage in terms of COD performance and deceleration abilities in agreement with McBurnie et al. (2021) (McBurnie et al., 2021). this could be due to the deceleration strategy before the change of direction moment such as a concept of ‘self-regulation’ tuning the approach velocity to better tolerate the kinetic energy (Hewit et al., 2011; Dos'Santos et al., 2018). This might also be due to a higher sprint momentum in male players compared to female players. Freitas et al. (2021) discussed that regarding the COD deficits, sprint momentum is a key outcome that potentially explains why men displayed higher values than females. Faster and heavier athletes need to apply higher braking forces through longer ground contact times to compensate for a higher sprint momentum (Freitas et al., 2021). It should be noted that almost half of the female soccer players were recruited from the first France league division (1–2 training sessions per day), which underlines the physical quality of the sample of female players tested in this study. If the player could not apply higher braking forces through longer ground contact times when the sprint momentum and approach velocity are greater before changing direction, consequently, lower COD performance (Dos'Santos et al., 2018). As a result, the individuals’ level and sports training experience may explain this discrepancy. Despite this difference in the level of play, the associations that have been established between the mechanical parameters of acceleration and deceleration and the ability to change direction remain relevant for both populations and provide new insights into the direction of training for male and female soccer players.

More importantly, the High/Fast and Low/Slow on the COD test in male players show a large difference in 10 m sprint time and P_max_ and BP_max_ differences concerning the mechanical parameters. This is another portion of the evidence that the metrics derived from the mechanical parameters profile obtained from acceleration and deceleration are not yet, capable of discriminating COD performance in male soccer players. On the other hand, female players with a Low/Slow COD performance show higher 10 m sprint time and much lower acceleration and deceleration mechanical parameters than those with High/Fast COD performance. This suggests that, in the female population, the differences in the level of play and training were very marked between the two groups and that the group of Low/Slow performers suffered from a substantial deficit of physical preparation, especially the sprint/acceleration training. This may explain the lack of significant multiple correlations between acceleration, deceleration capacities, and COD performance obtained in the present study. Despite this, it is worth noting that the current finding highlights a deficit of BP_max_ in Low/Slow COD female performers. The lower limb neuromuscular capacities of female players who might display Low/Slow COD performance seem to be highly deficient. Further studies may determine if the F-V profile could be advantageous in evaluating COD performance in a homogenous female soccer players group.

### Predictive COD performance models based on acceleration and deceleration abilities

Additionally, multiple linear regression shows a significant relationship between 10 m sprint time and HBF_max_, and Dec_max_, but only in male players, and the 10 m sprint time represented the higher regression coefficient (Figure 3). These findings are unsurprising, bringing further support to the paramount importance of the short sprint ability for COD performance compared to the deceleration capacity. In the basics of the previous study, the 10 m sprint time showed a higher correlation (r ≈ 0.7) with COD performance (Lockie et al., 2018), whereas the deceleration ability only indicated a moderate effect on the COD performance (i.e., Average horizontal Ground Reaction Force, *d* = -0.9, *p* = 0.05) (McBurnie et al., 2021). It could be assumed that the 505-test and the method used to calculate mechanical parameters during linear deceleration revealed some limitations. Clarke et al. (2020) showed that approximately 78% of athletes would either over or underestimate their deceleration ability during the COD test (Clarke et al., 2020). Indeed, the 505 test does not allow athletes to reach the maximum sprint speed and, therefore, to take advantage of the maximum braking capabilities. Thus, the athlete did not require excessive time to decelerate; in contrast, they still needed higher sprint performance to complete the COD task as it would be advantageous to enter and exit the COD as fast as possible. Additionally, the measurement of the deceleration performance, for now, is still challenging because of the influence of the maximal velocity reached before the deceleration (Graham-Smith et al., 2018). Meanwhile, considering the multiple factors that could impact COD performance, an optimal COD strategy/technique could also be a determining factor in the COD performance (Thomas et al., 2020a; McBurnie et al., 2021). It is, consequently, a more specific test on which physical abilities may have less influence than technical abilities.

### Limitation

Although our results indicated that certain mechanical parameters of acceleration and deceleration presented a weak to moderate correlation with COD performance, 10 m sprint time was still the best predictor of COD performance. Further prospective and experimental research are needed to evaluate the potential interest in the F-V profile in COD performance. Additionally, due to the high impact of lower limb neuromuscular capacity on the acceleration (Morin et al., 2015) and deceleration ability (Zhang et al., 2021a), it could be valuable to evaluate the lower limb maximal concentric and eccentric torques, RTD, muscular activation to explain these results. Moreover, considering the influence of the angle of the change of direction, it is suggested to consider multi-directional maneuver and technical/strategical components (e.g., penultimate and final foot contact, trunk inclination, or hip angle). It could also be noted that the current study analyzed COD performance on the right vs left sides rather than on the dominant (DL) vs non-dominant (NDL) leg. However, it should be noted that there was no difference in the COD performances between the DL and NDL, and it was also observed that almost all the subjects started the sprint with their left foot forward regardless of their dominant foot. For these reasons, the analysis of the left-right side seems more relevant.

## Conclusion

The present study aimed to estimate the magnitude of the association between different mechanical parameters derived from acceleration and deceleration and COD performance in male and female soccer players. Considering the large number of factors influencing COD performance, it seems that the short sprinting performance (i.e., 10-m) plays a critical role in determining COD performance. Furthermore, it seems that the computation of F-V parameters remains less reliable than time measurements but could provide some additional information to assess an athlete’s ability to change direction. Although certain mechanical parameters of deceleration presented a moderate correlation with COD performanceu, it should be noted that these mechanical parameters have different influences on the COD performance in male and female soccer players.

### Practical application

Based on the current finding, the ability to perform 10 m sprint times is critical/necessary to achieve high COD performance. Training programs should enhance fast sprint capability through heavy resistance and/or high-velocity training exercises. In addition, special muscle training, such as the eccentric force of quadriceps, might contribute to the high braking force production during the deceleration task (Zhang et al., 2021a). Moreover, the results obtained for women demonstrate the need to develop physical preparation even for amateurs.

## Data Availability

The raw data supporting the conclusions of this article will be made available by the authors, without undue reservation.
